# Trends in the shortfall of English NHS general practice doctors: repeat cross sectional study

**DOI:** 10.1136/bmj-2024-083978

**Published:** 2025-09-17

**Authors:** Luisa M Pettigrew, Aamena Valiji Bharmal, Soraya Akl, Josephine Exley, Luke N Allen, Irene Petersen, David A Cromwell, Nicholas Mays

**Affiliations:** 1 Department of Health Services Research and Policy, London School of Hygiene and Tropical Medicine, London WC1H 9SH, UK; 2 The Health Foundation, London, UK; 3 School of Primary Care and Public Health, Imperial College London, London, UK; 4 Global Primary Care and Future Health Systems, Nuffield Department of Primary Care Health Sciences, University of Oxford, Oxford, UK; 5 Department of Primary Care and Population Health, University College London, London, UK; 6 Department of Clinical Epidemiology, Aarhus University, Aarhus, Denmark

## Abstract

**Objectives:**

To compare the numbers and characteristics of English general practitioner doctors (GPs) across publicly available data sources, and to examine trends in GP numbers relative to population growth and the specialist medical workforce in England.

**Design:**

Repeat cross sectional study.

**Setting:**

Three national data sources, England, 2012-24: General Medical Council (GMC) GP and specialist registers; NHS England GP Performers List; and NHS England’s General Practice Workforce and NHS Workforce Statistics datasets.

**Participants:**

All GMC licensed, fully qualified GPs in England.

**Main outcome measures:**

Differences over time in total numbers and GP characteristics. Changes in the difference between GMC and NHS general practice GP numbers and characteristics, and analysis of trends relative to population size and equivalent data on specialist doctors.

**Results:**

As of 31 December 2024, 58 548 GPs were listed on the GMC GP register, 55 958 on the Performers List, but only 38 626 by headcount and 28 197 by full time equivalent GPs in NHS general practice. Between 2015 and 2024, on average, for every five additional GPs licensed by the GMC, NHS general practice lost one full time equivalent GP each year. As a result, the proportion of GMC licensed GPs not working in NHS general practice increased from 27% (13 492) in 2015 to 34% (19 922) in 2024 by headcount and from 41% (20 210) to 52% (30 351) by full time equivalent GPs. Differences were greatest among female GPs, younger GPs, UK qualified GPs, and GPs in London and the South East of England. In contrast, between 2015 and 2024, for every five additional GMC licensed specialist doctors, the NHS gained 4.3 full time equivalent consultants. Taking population growth into account, the number of NHS patients for each full time equivalent GP in NHS general practice increased by15%, whereas the number of patients for each full time equivalent NHS consultant fell by 18%. By the end of 2024, there were twice as many NHS patients for each full time equivalent NHS general practice GP (2260) than for each full time equivalent NHS consultant (1092).

**Conclusion:**

The growing difference between GMC licensed GPs and those working in NHS general practice is in contrast with trends among specialists. This shift is occurring despite rising patient demand and policy commitments to strengthen primary care. Addressing the underlying reasons for workforce attrition in NHS general practice is critical to achieving the government’s stated goals of strengthening community based care and shifting the focus of care from treatment to prevention.

## Introduction

The provision of general practitioner (GP) doctors in England’s NHS general practices has failed to keep up with population growth and needs in recent years.^1^ ^2^ ^3^ Despite the importance of primary care for all health systems, this challenge is not unique to England and has resulted in multiple government promises to increase the general practice workforce.^4^ ^5^ ^6^ ^7^ ^8^ ^9^ ^10^ ^11^ Efforts in England have largely focused on increasing postgraduate NHS GP trainees.^10^ ^11^ ^12^ While trainee numbers have doubled since 2015, and an expansion of other roles in general practice has occurred, concerns about the provision of GPs in NHS general practice remain, and calls have been made to prioritise research into the primary care workforce, both in the UK and internationally.^10^ ^13^ ^14^

To work as a GP, either in the NHS or privately, doctors must meet the requirements of the General Medical Council (GMC, the UK’s regulatory body for doctors) to be included on its GP register. Doctors need to complete postgraduate GP training and maintain a GMC licence to practise. To maintain a GMC licence, GPs must pay an annual fee (£461 (€532; US$619) in April 2025) and revalidate every five years.^15^ Revalidation requires GPs to practise, on average, a minimum of one session (4.17 hours) a week and complete an annual peer led appraisal of their work and performance.^16^ Also, to be eligible to work in the NHS, GPs need to be included on the NHS England GP Performers List,^17^ which requires identity, training, appraisal, medical indemnity, language, and police checks. In England, the Performers List is managed by Primary Care Support England on behalf of NHS England. A minimum of 10 years is needed to train to become a GP, and the total undergraduate and postgraduate investment was estimated at £430 540 in 2022 in the UK.^18^

With the aim of informing primary care workforce policy and better understanding the shortfall between GPs in NHS general practice and the total number of GPs in England, we examined the differences over time in the number and characteristics of GPs on the GMC register, Performers List, and in NHS general practice, and compared the trends relative to population size as well as equivalent specialist, mainly secondary care based, doctor data.

## Methods

### Data sources

We examined general practice data from the three organisations that collect and publicly report national workforce statistics in England.

1. The GMC GP register. A list and count of GPs with a licence to practise who have completed the necessary training and meet regulatory requirements to be appointed as a GP in the UK, both in the NHS and privately.^7^ ^19^ ^20^
2. NHS England GP Performers List. A list of GPs approved by Primary Care Support England to provide care in the NHS (in general practice or elsewhere, eg, walk-in centres or prisons) or the armed services in England.^17^ ^21^
3. NHS England General Practice Workforce datasets. Collection of monthly online reports by nominated practice managers on staff working in NHS general practices.^10^

**Fig 1:**
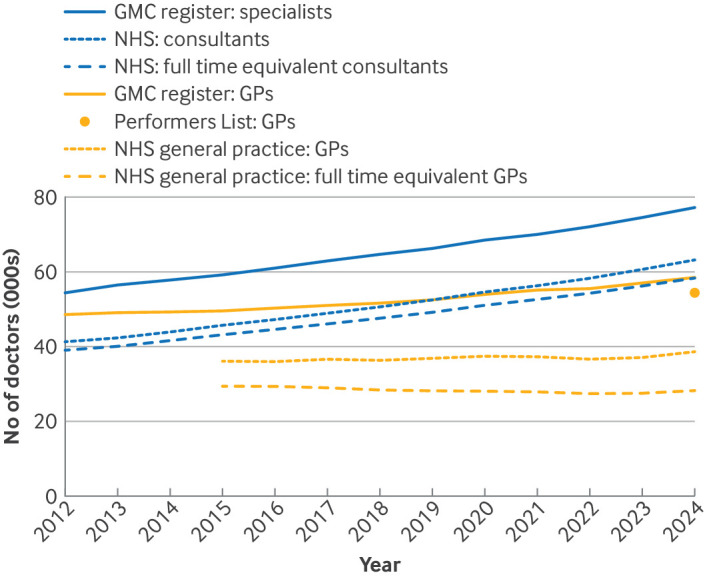
Total number of licensed, fully qualified (ie, excluding trainees) general practitioners (GPs) in England from the General Medical Council (GMC) GP register, NHS England GP Performers List, and NHS England General Practice Workforce datasets (by headcount and full time equivalent GPs). Total number of licensed specialists in England from the GMC specialist register and consultants working in NHS trusts or other core organisations, excluding primary care from NHS England Workforce Statistics (by headcount and full time equivalent consultants). The Performers List only allows data to be downloaded in full for the current day and therefore total headcount of GPs on the Performers List is reported for 31 December 2024

For comparison with the number of specialist doctors, we examined data from the GMC specialist register with a licence to practise. The GMC specialist register is a list of doctors who have completed the necessary training and meet regulatory requirements to be appointed as a consultant in the UK, both in the NHS and privately.^22^ We also assessed the number of consultants working in NHS trusts and other core organisations in England (excluding primary care) from data from NHS England Workforce Statistics.^23^ We acknowledge that GPs are specialists in general practice, but we use the term specialist here to refer to doctors who qualify to be in the GMC specialist register and therefore work as consultants.^24^ Supplementary figure 1a and figure 1b present the hierarchies of the populations covered by the different data sources for GPs and specialist doctors. We used datasets from NHS England on patients registered at a GP practice to calculate the number of patients for each doctor.^25^

### Data extraction and preparation

Data were extracted from each organisation’s websites by one author (LMP) and cross checked by one of two other authors (AVB or SA). Supplementary table 1 provides a description of the data publicly available on GPs from each source.

#### GMC registers

Data from 31 December for every year between 2012 and 2023 were extracted from *The state of medical education and practice in the UK: Workforce report 2024*.^7^ ^22^ Data included the total headcount of GPs and specialist doctors with a licence to practise, as well as personal characteristics of the GPs: gender, as reported in the national data sources, age band, and place of primary medical qualification. GP data for 2024 were taken from the GMC Data Explorer on 31 December 2024, including which of the seven NHS regions the GP’s home postcode corresponded to, but excluding GP age bands, which were not available.^20^ Data from the GMC specialist register for 2024 were accessed later, on 18 March 2025.

#### Performers List

The Performers List only allows data to be downloaded in full for the current day. Historical data are not publicly available. Therefore, we were limited to reporting the total headcount of GPs on the Performers List at one time point, 31 December 2024.^17^ ^21^ We extracted all listed fully qualified GPs, which included GPs in the armed services. We excluded GPs who were suspended.

#### NHS General Practice Workforce and NHS Workforce Statistics

Data from 31 December for each year between 2016 and 2024 were used for comparison with GMC derived figures. In 2015, no GP NHS data were published in December, and therefore we used data from 30 September 2015.^10^ ^23^ NHS workforce data extracted included the total headcount and full time equivalent number of fully qualified GPs (ie, including long term locums, but excluding GP trainees) in NHS general practice and, for comparison, consultants working in NHS trusts and other core NHS organisations in England. GP data by gender, age band, place of primary medical qualification, and NHS region in England where the GP was reported working, were extracted from General Practice Workforce Bulletin tables.^10^ GP numbers grouped by both age band and gender were calculated from NHS General Practice Workforce individual GP level data, by adding full time equivalent totals and unique GP headcounts. Full time equivalent refers to the proportion of full time contracted hours that the post holder is contracted to work. NHS England defines full time as 37.5 hours/week for a fully qualified GP.^10^ A GP’s place of primary medical qualification by full time equivalent is not published. Short term, ad hoc, GP locums, and GPs employed by primary care networks working in NHS general practice are reported separately by NHS England and here.

#### NHS patients registered at a GP practice

We used the total number of patients registered in general practice in England on 1 January and divided this value by the number of doctors on 31 December of the preceding year to calculate the number of patients for each doctor. Data were available from 2014 to 2025.^25^

### Statistical analysis

We used descriptive statistics to compare total numbers, number of registered patients for each doctor, and GP characteristics (gender, age band, country of qualification, and region in England) over time. We report the coefficient for the absolute change for each year, estimated from linear regression trend analyses, with 95% confidence intervals (CIs). We used these coefficients to calculate simplified ratios to enable comparison between changes in GMC and NHS workforce numbers. We report the number and percentage of GMC licensed doctors not appearing in the NHS General Practice Workforce data by headcount and full time equivalent hours. We also report changes over time in full time equivalent to headcount proportions of GPs in NHS general practice. We present GMC and NHS consultant data from when the data were available. We used 2015 as the baseline for comparing GMC and NHS General Practice Workforce figures because 2015 was the first common time point. The final comparison time point for GMC and NHS was December 2024, except for GPs grouped by both gender and age band, which was December 2023 because of GMC data availability. Stata 18 was used for regression analysis. The supplementary material shows data sources, data used, Stata code, and results.

NHS England’s Primary Care Workforce Data and Analytics team, Primary Care Support England’s online portal for queries, and the GMC’s Insight and Research team were consulted when queries arose. Findings are reported based on the Reporting of Studies Conducted Using Observational Routinely Collected Data (RECORD) guidelines.^26^

### Patient and public involvement

This paper is part of a wider research project examining the effect of inspections on the quality of general practice where patients and the public have been involved in the design and undertaking of the study. Several drafts of this paper were reviewed by a patient with research expertise and who is a member of their general practice’s patient participation group.

## Results

### Comparing total numbers of GPs and specialist doctors

Figure 1 and supplementary table 2 show the total number of GPs and specialist doctors over time. Between 2015 and 2024, the number of GPs licensed by the GMC increased from 49 574 to 58 458, averaging an extra 985/year (95% CI 884 to 1086). In contrast, GMC licensed specialists increased at almost twice this rate, from 59 172 to 77 299, averaging an extra 1948/year (95% CI 1832 to 2063). Meanwhile, in NHS general practice, the headcount of fully qualified GPs only increased by, on average, 207/year (95% CI 82 to 332), from 36 082 to 38 626. Adjusting for reported working hours, full time equivalent GPs decreased at a rate of 199/year (95% CI −295 to −102), from 29 364 to 28 197. The number of NHS consultants increased from 45 655 to 63 244, an average rate of 1929/year (95% CI 1821 to 2037), with full time equivalent consultants also increasing by 1674/year (1602 to 1745) from 43 176 to 58 382 (supplementary table 2). Hence for every five additional GMC licensed GPs, NHS general practice only gained 1.05 GPs by headcount, and lost 1.01 GPs by full time equivalent GP. In contrast, for every five additional GMC licensed specialists, the NHS gained 4.95 consultants by headcount and 4.3 consultants by full time equivalent, resulting in double the number of NHS consultants than NHS general practice GPs by December 2024.

The average full time equivalent GPs to headcount proportion decreased from 0.81 to 0.73 between 2015 and 2024 for GPs in NHS general practice (supplementary table 3). In contrast, fewer NHS consultants worked part time and the full time equivalent to headcount ratio only decreased marginally from 0.95 to 0.92. Hence GPs were more likely to be reported as working part time in NHS general practice, and the likelihood increased at a faster rate than for NHS consultants.

The proportion of GMC licensed GPs not working in NHS general practice increased from 27% (13 492) in 2015 to 34% (19 922) in 2024 by headcount and from 41% (20 210) to 52% (30 351) by full time equivalent GPs. In contrast, the proportions of GMC licensed specialists not working as an NHS consultant decreased from 23% (13 517) to 18% (14 055) by headcount and from 27% (15 996) to 24% (18 917) by full time equivalent consultants (supplementary table 2). This finding means that in 2024, 2.1 GMC licensed GPs are needed to have one full time equivalent GP in NHS general practice, whereas only 1.3 GMC licensed specialists are needed to have one full time equivalent NHS consultant (fig 1 and supplementary tables 2 and 3).

### Added insight from Performers List

In 2024, 55 958 fully qualified GPs were on the Performers List (including 200 GPs working in the armed services and excluding 31 suspended GPs). The difference of 2590 GPs between the GMC register and Performers List represents GPs with an active licence to practise but who have not applied or have not been approved to work in an NHS or armed services setting in England (supplementary table 2). The difference of 17 332 by headcount and 27 761 by full time equivalent GPs between the Performers List and NHS General Practice Workforce data represents GPs who are not working in NHS general practice (although they might be working elsewhere in the NHS), despite being eligible to do so.

### Other GPs in NHS general practice

In December 2024, as well as the numbers reported above, there were 1299 ad hoc GP locums by headcount, 292 by full time equivalent, who were reported separately from the main general practice workforce dataset.^10^ Also, there were 368 GPs, 189 by full time equivalent, who were employed by primary care networks and likely to be based in NHS general practice.^27^ Combining these figures would represent an additional 1667 (4%) GPs, 481 (2%) by full time equivalent, in NHS general practice.

### Numbers of registered patients for each GP and specialist doctor

The number of patients registered with an NHS general practice increased by 11% from 57 540 101 to 63 724 968 between 1 January 2016 and 1 January 2025. Because of this increase, alongside the decrease in the numbers of full time equivalent GPs in NHS general practice, the average number of patients for each full time equivalent GP in NHS general practice increased by 15% from 1960 to 2260 (supplementary table 4). In contrast, because of the increase in NHS consultants, the number of registered patients for each full time equivalent NHS consultant decreased by 18%, from 1333 to 1092 (supplementary figure 2 and supplementary table 4).

### Comparing GMC licensed and NHS general practice GP characteristics

#### Gender

**Fig 2:**
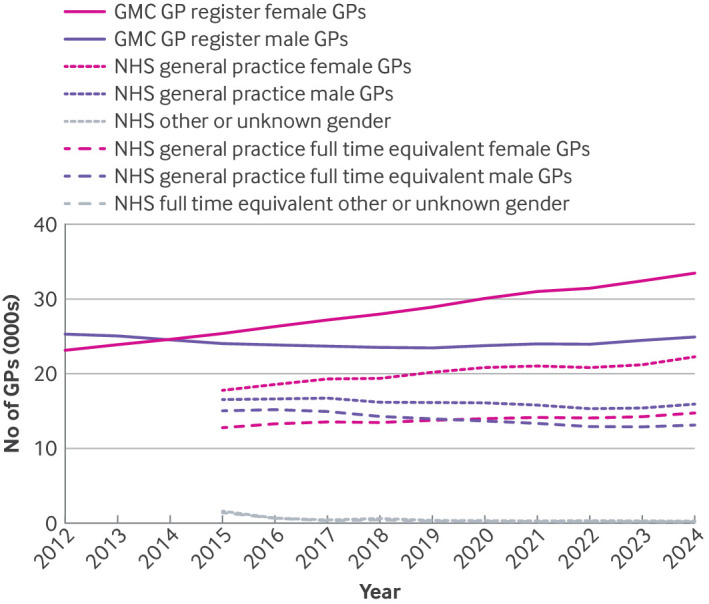
Total number of licensed, fully qualified general practitioners (GPs) from the General Medical Council (GMC) GP register and reported in NHS general practice (by headcount and full time equivalent GPs) in England by female or male gender. NHS other or unknown=other or unknown gender of GPs working in NHS general practice

In 2014, the number of GMC licensed female GPs overtook the number of male GPs, and in 2020, total full time equivalent female GPs also overtook full time equivalent male GPs in NHS general practice (fig 2). Between 2015 and 2024, GMC licensed female GPs increased on average by 892 GPs/year (95% CI 854 to 931, supplementary table 5). Less than half of this annual increase was seen in the number of female GPs in NHS general practice (438/year, 95% CI 348 to 528), and less than a fifth by full time equivalent (178/year, 138 to 218). The number of GMC licensed male GPs increased marginally between 2015 and 2024 (92/year, 95% CI 0 to 185). A marked decrease, however, in male GPs in NHS general practice by headcount (−135/year, 95% CI −203 to −67) and more so by full time equivalent GPs (−282/year, −350 to −215) was found (supplementary table 5).

In 2024, the full time equivalent to headcount ratio was 0.66 for female GPs and 0.82 for male GPs (supplementary table 3). Although female GPs were more likely to work part time, the difference in the proportion working part time in NHS general practice between men and women diminished over the eight year period because of a greater reduction in full time equivalent hours by male GPs.

The proportion of GMC licensed female GPs not working in NHS general practice increased from 30% in 2015 by headcount to 33% by 2024; by full time equivalent GPs, this proportion increased from 50% to 56% (supplementary table 5). For GMC licensed male GPs, the proportion not in NHS general practice increased from 31% to 36% by headcount and from 37% to 47% by full time equivalent GPs. In 2024, the largest absolute difference between GMC licensed and NHS general practice GPs was for female GPs, both by headcount (11 215) and full time equivalent GPs (18 752) (supplementary table 5).

#### Workforce by age band and gender

**Fig 3:**
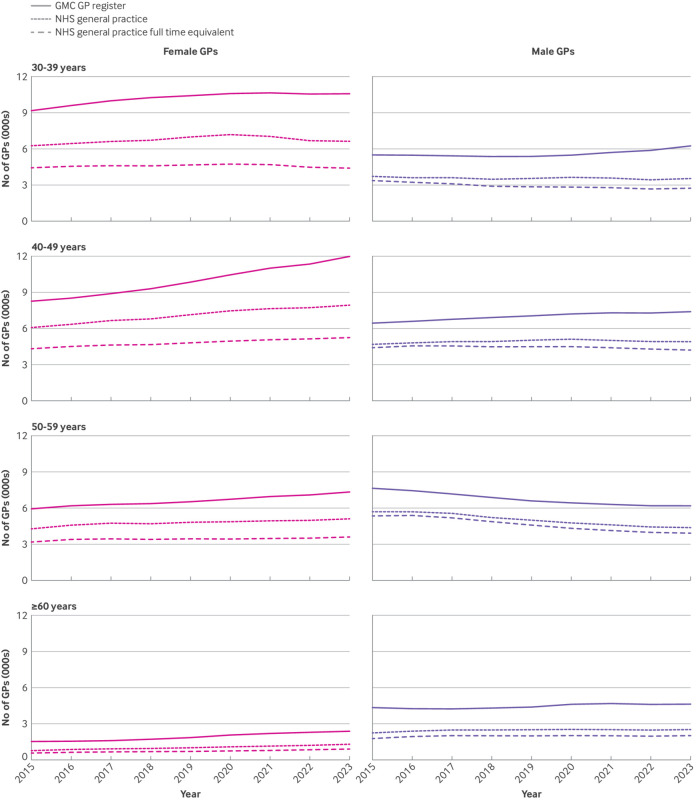
Total number of licensed, fully qualified general practitioners (GPs) from the General Medical Council (GMC) GP register and reported in NHS general practice (by headcount and full time equivalent) in England by age band and gender

Figure 3 summarises trends by age band and gender. GMC licensed female GPs aged 40-49 year represented the largest cohort of GPs in England by 2023 (12 018), closely followed by female GPs aged 30-39 years (10 604). These two cohorts, however, also showed the largest and a widening gap between GMC licensed and NHS general practice GPs. We found, on average, an additional 166/year (95% CI 90 to 242) GMC licensed GPs aged 30-39 years and an additional 481/year (441 to 521) aged 40-49 years, but in NHS general practice these figures were only 59/year (−22 to 140) by headcount with a negligible fall in full time equivalent GPs (−0.34/year, −39 to 38) for those aged 30-39 years (supplementary table 6a). For those aged 40-49 years, less than half of the rise was seen (240/year, 95% CI 209 to 270) by headcount and less than a quarter (114/year, 103 to 125) by full time equivalent GPs in NHS general practice (fig 3).

Between 2015 and 2023, the proportion of GMC licensed female GPs not in NHS general practice increased from 32% to 37% by headcount and from 52% to 58% by full time equivalent for those aged 30-39 years, and from 27% to 34% by headcount and 48% to 56% by full time equivalent for those aged 40-49 years (supplementary table 6a). Despite an overall 14% rise in GMC licensed male GPs aged 30-39 years between 2015 and 2023, a 19% reduction was seen in NHS general practice.

As the number of female GPs aged >50 years has risen, the number of male GPs aged >50 years has decreased. GMC licensed male GPs aged 50-59 were less likely to work part time in NHS general practice compared with their female counterparts. Female GPs aged >60 years, however, if they retain their GMC licence, were more likely to work in NHS general practice. Supplementary tables 6a and 6b, and supplementary figure 3 report all results grouped by age bands.

#### Place of primary medical qualification

**Fig 4:**
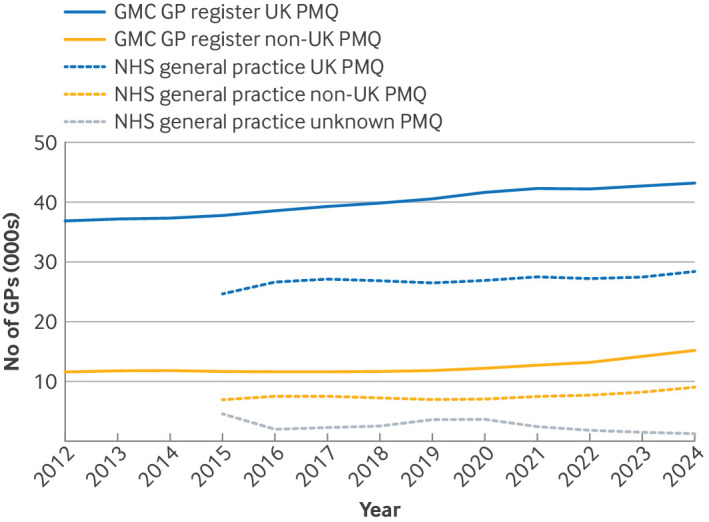
Total number of licensed, fully qualified general practitioners (GPs) from the General Medical Council (GMC) GP register and reported in NHS general practice (by headcount and full time equivalent GPs) in England by place of primary medical qualification (PMQ). UK=place of primary medical qualification from the UK; non-UK=place of primary medical qualification from outside the UK; NHS unknown=unknown place of primary medical qualification for GP working in NHS general practice

Between 2015 and 2024, UK medical graduates accounted for, on average, 76% of GMC licensed GPs and 73% of GPs in NHS general practice (supplementary table 7a). During this period, the number of GMC licensed UK qualified GPs increased by 613/year (95% CI 528 to 698), whereas NHS general practice GPs increased by less than half this rate (258/year, 104 to 412, supplementary table 7b). Similarly, the number of GMC licensed GPs who qualified outside the UK increased by 372/year (95% CI 223 to 520), but less than half of this increase was seen in NHS general practice (155/year, 35 to 274). Some of the increases in NHS general practice GPs were a result of the reallocation of GPs whose place of primary medical qualification had been previously classed as unknown (fig 4 and supplementary table 7b). In 2024, the largest discrepancy between GMC licensed and NHS general practice GPs was for UK graduates (14 827, 34% of UK qualified GPs), but the greatest relative discrepancy was for non-UK qualified GPs (6168, 40% of non-UK qualified GPs).

#### Regional differences in number of registered patients for each GP in 2024

**Fig 5:**
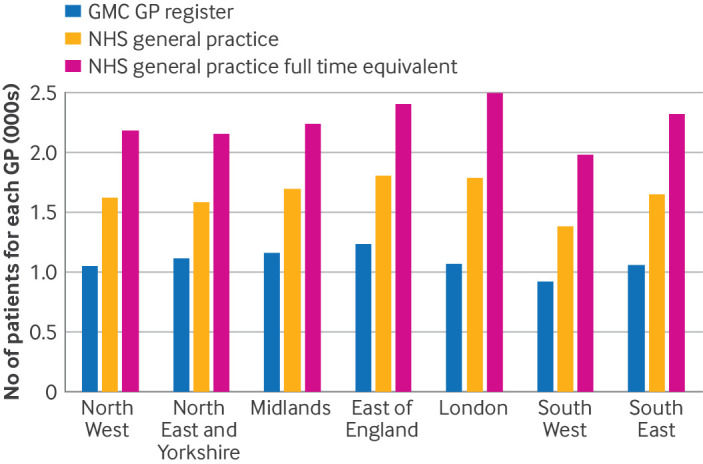
Comparison of the average number of NHS registered patients for each licensed, fully qualified general practitioner (GP) from the General Medical Council (GMC) GP register and reported in NHS general practice (by headcount and full time equivalent GPs) in England by region, December 2024

In 2024, London had the highest number of NHS registered patients for each full time equivalent NHS general practice GP (2496). Differences in the average number of NHS registered patients for each GMC and NHS general practice GP were also greatest in London, followed by the South East of England. The smallest differences were in the North East and Yorkshire region. The South West had the fewest number of patients (1984) for full time equivalent NHS general practice GP. GPs in the South West were also most likely to work part time in NHS general practice, whereas GPs in the Midlands were the least likely, with full time equivalent ratios of 0.70 and 0.76, respectively (fig 5 and supplementary tables 8 and 9).

## Discussion

### Principal findings

We found that, on average, for every five additional GMC licensed GPs, NHS general practice lost one full time equivalent GP each year for the period 2015-24. As a result, the overall proportion of GMC licensed GPs not working in NHS general practice has risen, now representing more than a third by headcount count and more than half by full time equivalent GPs. In the context of population growth, during the same period, the number of NHS registered patients for each full time equivalent GP in NHS general practice increased by 15%. In contrast, for every five additional GMC licensed specialist doctors, the NHS gained 4.3 full time equivalent consultants, and the number of patients for each full time equivalent NHS consultant decreased by 18%, resulting in double the number of NHS patients for each GP in NHS general practice than for each NHS consultant.

The general practice workforce has become increasingly reliant on female GPs and GPs aged 40-49 years. The discrepancy between the number of GMC licensed and NHS general practice GPs was greater for women than men. By age band, the greatest difference was in those aged 30-39 years by headcount, with a trend suggesting that most newly qualified GPs are not entering the NHS general practice workforce or leaving within the first 10 years. The fastest rate of decline in NHS general practice was seen in male GPs aged 50-59 years. The number of GMC licensed UK medical graduates not in NHS general practice was greater than the number of international medical graduates, but the proportion was higher for international medical graduates. The average number of registered patients for each full time equivalent NHS general practice GP was highest in London, where the greatest differences were also found between the number of NHS patients for each GMC licensed and NHS general practice GPs. 

### Strengths and limitations of this study

Our study compared three national sources of GP workforce data in England, building on previous work in this area.^28^ ^29^ ^30^ ^31^ Our results have provided unique insights into how GP trends and characteristics differ between these sources. The study also compared trends in specialist doctors, as well as reported on the number of NHS patients for each doctor.

Limitations to the datasets exist. Firstly, the lack of published time series data for the Performers List prevented longitudinal analysis. Secondly, GP data submissions to NHS England require practices to log onto the National Workforce Reporting Service to update information. On average, 1% of GPs by headcount and 2% by full time equivalent (including trainees) GPs had to be estimated by NHS England because of missing data between September 2015 and December 2024.^10^ Thirdly, although workforce submissions are mandatory and NHS England reports the last time individual practices updated their workforce data, how many records are not up to date is not reported.^32^ ^33^ Fourthly, analysis of the National General Practice Worklife Surveys found that GPs were working, on average, 49.2% more for each contracted session in 2021.^34^ Full time equivalent hours reported by NHS England, however, most likely reflect contracted hours and therefore underestimate the true working hours of GPs.^35^ Fifthly, in mid-2023, 4.9 million (8%) more patients were registered in NHS general practice than estimated by the population based census of the Office of National Statistics.^36^ The increased numbers in the NHS list is attributed to reasons such as delayed de-registrations and duplicate registrations, but under-coverage also exists because of, for example, unregistered migrants and existing patients being inappropriately removed under the no contact criteria.^37^ These differences generate some uncertainty about which population count to use.^38^ Finally, regional analysis assumed that GPs work in the same region that they live, which might not always be the case.

### Possible reasons for discrepancies between GP data sources

#### GMC licensed GPs not on the Performers List

Reasons why GMC licensed GPs are not be on the Performers List might be because GPs are: in the process of joining the Performers List; in the process of leaving the GMC register; have recently moved outside of England without informing the GMC; are working or training as a specialist (while also licensed as a GP); or are working only in private practice. Since the covid-19 pandemic, a rise in demand for private GPs has been reported by the media, and the expansion of online general practice services has made it easier to work privately.^39^ Third party estimates suggest that 2000-3000 GPs currently work in private practice.^40^ What proportion of GPs might combine private practice work with NHS work, however, is unclear, as is how this proportion has changed over time.

#### GMC licensed GPs on the Performers List but not in NHS general practice

The discrepancy between the Performers List and NHS England’s GP headcount might be explained, in part, by NHS England General Practice Workforce data not capturing GPs working within the NHS but outside traditional NHS general practice, such as in prisons, army bases, walk-in centres, or other alternative NHS settings.^41^ ^42^ That these roles would account for more than a third of GP related NHS activity seems unlikely however, unless most of the GPs in this group are undertaking small amounts of such work (eg, ad hoc shifts in NHS walk-in centres). In 2019, NHS England tried to capture statistics on GPs working in alternative NHS settings but stopped because of concerns with data quality.^41^ GPs only working as ad hoc locums or employed by primary care networks are also not reported within the main NHS General Practice Workforce datasets. Despite a recently publicised increase in GP recruitment through primary care networks, however, they still represent a small proportion of full time equivalent GPs.^43^

Another explanation for the discrepancy could be because GPs only intermittently appear in NHS general practice data (eg, only working some months of the year) when on parental or sick leave, for example. GPs might also remain on the Performers List despite no longer working in the NHS in England or they might be looking for work in NHS general practice.^42^ The difference between the Performers List headcount and full time equivalent GP figures in NHS general practice might also be explained by GPs combining NHS general practice work with other general practice related work (eg, teaching, research, leadership, or management) in the NHS or elsewhere (eg, private practice), as a portfolio career, or working part time, or both.^44^ ^45^

### Implications for research and policy

#### Understanding how GPs spend their time

Currently no data exist on what GMC licensed GPs do outside of NHS general practice. More data on this area are needed to understand patterns of general practice workforce distribution which might relate to weaknesses in NHS general practice. We suggest that the GMC considers collecting and publishing information about the type and quantity of work undertaken by GPs as part of the annual retention fee process or by annual surveys. Medical indemnity organisations could be asked to publish non-identifiable data on the type and quantity of work that is done by the GPs that they insure. Qualitative research could target GP groups that seem more likely to not work in NHS general practice, while maintaining their licence to practise, to better understand their reasons for this. Within NHS general practice, collection of full time equivalent hours could be improved by inviting individual GPs to cross check and approve data on working hours submitted on their behalf to NHS England.

#### Closing the gap

The gap between GMC licensed and NHS general practice GPs in 2024 represented an estimated £8.6 billion investment in training by headcount and £13.1 billion by full time equivalent GPs lost from NHS general practice, although this figure will be less for the UK after adjusting for international medical graduates.^18^ Comparison with specialists suggests that workforce planning has intentionally, or otherwise, prioritised the secondary care medical workforce.^12^ This finding is at odds with international evidence indicating that additional investment in primary care, including GPs, gives better population health, more equitable outcomes, and more efficient health systems compared with the equivalent spent on secondary care.^46^ ^47^ ^48^ ^49^ ^50^

Previous research examining reasons for GPs leaving NHS general practice or reducing working hours frequently describe push factors, such as having insufficient time with patients, loss of continuity of care, administrative burdens, unsustainable workloads, feeling unsupported, and being burnt out.^51^ ^52^ ^53^ ^54^ ^55^ ^56^ ^57^ ^58^ More recently, problems with pensions and income have also been cited as reasons.^59^ Recruitment and retention problems in primary care are not unique to England, but a 2022 international comparative study found that GPs in the UK were the most likely to report planning to stop seeing patients regularly in the next 1-3 years.^14^ ^60^

Looking at what can be done to retain GPs leaving NHS general practice or reducing their working hours because of parental and other caring roles is particularly relevant with an increasing number of women in the workforce because typically, a larger proportion of these competing responsibilities fall on women.^45^ ^61^ The diminishing proportion of GMC licensed GPs aged 30-39 years in NHS general practice needs to be looked at urgently because the future of NHS general practice depends on this group. Research into the pull factors of alternative career paths would help inform policy to improve working conditions in NHS general practice. Understanding labour elasticity is also important (ie, if income is increased, would GPs be more likely to reduce their working hours, rather than maintain or increase their hours, and how would this behaviour vary between GPs with different characteristics?).^62^ Barriers to entering NHS general practice need to be looked at, particularly with recent reports of GPs unable to find NHS general practice work despite a well reported need for these GPs.^63^ ^64^ This situation has been attributed to earmarked funding streams incentivising the employment of other direct patient care roles in general practice, such as pharmacists, physician associates, and paramedics, rather than GPs.^63^ ^64^

Difficulties in international medical graduates accessing work visas on completing their training in the UK have also been highlighted as a problem.^57^ ^65^ This problem is particularly relevant because the doubling of GP trainees since September 2015 has largely been a result of international medical graduate recruitment who represented 52% of GP trainees by August 2024 (*v* 25% in December 2018).^10^ The dependence of the NHS on international medical graduates raises ethical questions, particularly when recruited from countries with greater workforce shortfalls than the UK, when often asked to work in the most challenging of English contexts without adequate support, and when these graduates seem more likely to not work in NHS general practice than UK graduates.^66^ ^67^ Regional differences might be reflective of greater opportunities for portfolio careers and private practice, taking GPs out of NHS general practice in London and the South East. Responding to these variations in GP provision will require careful policy design to attract GPs to the areas of greatest need.^68^ ^69^

### Conclusions

A growing difference has emerged between the number of GMC licensed GPs and those actively working in NHS general practice in England, with more than a third by headcount and more than half by full time equivalent GPs not appearing in NHS General Practice Workforce data in December 2024. This trend contrasts with patterns seen among specialists and is occurring despite rising patient demand and policy commitments to strengthen primary care. Dealing with the factors driving an increasing proportion of GPs to retain their licence but not practise in NHS general practice is essential to strengthening community based care and improving the effectiveness of NHS workforce planning.

**What is already known on this topic**

General practice in England has persistent recruitment and retention challenges
Rising patient-to-general practitioner (GP) ratios reflect growing demand and strain on the workforce

**What this study adds**

Between 2015 and 2024, on average, for every five additional General Medical Council (GMC) licensed GPs, NHS general practice lost one full time equivalent GP each year
Differences in the numbers of GMC licensed GPs and those working in NHS general practice were greatest among female GPs, younger GPs, UK qualified GPs, and GPs in London and the South East
The number of NHS patients for each full time equivalent GP in NHS general practice was twice that of full time equivalent NHS consultants by the end of 2024, highlighting a widening imbalance between primary and secondary care medical workforce capacity

## Data Availability

The code used to analyse the data in the paper can be found in the supplemental files. The data underlying the findings in this paper are openly and publicly available and can be found in the links provided in the supplementary material.

## Web extra

Extra material supplied by authors

Appendix 1: Supplementary materials

Appendix 2: Data sources, Stata code, and results

## Notes

Contributors: All authors made substantial contributions to the conception or design of the work, or the acquisition, analysis, or interpretation of data for the work; drafting the work or reviewing it critically for important intellectual content; final approval of the version to be published; and are accountable for all aspects of the work in ensuring that questions related to the accuracy or integrity of any part of the work were appropriately investigated and resolved. LMP is the study guarantor. The corresponding author attests that all listed authors meet authorship criteria and that no others meeting the criteria have been omitted.

Transparency: The lead author (the manuscript’s guarantor) affirms that the manuscript is an honest, accurate, and transparent account of the study being reported; that no important aspects of the study have been omitted; and that any discrepancies from the study as planned (and, if relevant, registered) have been explained

Dissemination to participants and related patient and public communities: We plan to present our findings at relevant national and international forums and conferences to reach relevant stakeholders. We also plan to use social media outlets to disseminate findings.

Provenance and peer review: Not commissioned; externally peer reviewed.
