# Trends in the shortfall of English NHS general practice doctors: repeat cross sectional study

**DOI:** 10.1136/bmj.r1997

**Published:** 2025-09-23

In this paper by Pettigrew and colleagues (*BMJ* 2025;390:e083978, doi:10.1136/bmj-2024-083978, published 17 September 2025), the first name of the last author Mays did not appear in the online version, owing to a tagging error. This has been corrected.

