# Supplementary material for: Trends in the shortfall of English NHS general practice doctors: repeat cross sectional study
Source: BMJ. 2025 Sep 17;390:e083978. doi: 10.1136/bmj-2024-083978 (PMC12442227; doi:10.1136/bmj-2024-083978)
Supplement: Supplementary file 1 — Appendix 1: Supplementary materials [file petl083978.ww1.pdf]

Trends in the shortfall of English NHS general practice GPs: a repeat cross-sectional study

BMJ

Luisa M Pettigrew, Aamena Valiji Bharmal, Soraya Akl, Josephine Exley, Luke N Allen, Irene Petersen, David Cromwell, Nicholas Mays

Contents

List of abbreviations ... 2
Supplementary Figures 1a & 1b - Relationship between data sources ... 3
Supplementary Table 1 - Data on general practitioners available ... 4
Supplementary Table 2 - Total number of general practitioners and specialist doctors ... 7
Supplementary Table 3 - Full-time-equivalent to headcount proportions ... 8
Supplementary Figure 2 - NHS registered patients-per-doctor ... 9
Supplementary Table 4 - NHS registered patients-per-doctor ... 10
Supplementary Table 5 - General Practitioners by gender ... 11
Supplementary Table 6a - General Practitioners by age-band and gender ... 12
Supplementary Table 6b - General Practitioners by age-band only ... 14
Supplementary Figure 3 - General Practitioners by age-band ... 15
Supplementary Table 7a - General Practitioner proportion by place of primary medical qualification ... 16
Supplementary Table 7b - General Practitioners by place of primary medical qualification ... 17
Supplementary Table 8 - Patients-per-FTE general practitioner by region ... 18
Supplementary Table 9 - Full-time-equivalent to headcount proportion by region ... 19

List of abbreviations

GP	General Practitioner
GMC	General Medical Council
NHS	National Health Service
NHSE	National Health Service England
FTE	Full-time-equivalent
HC	Headcount
PCSE	Primary Care Support England
ONS	Office for National Statistics
PMQ	(Place of) Primary Medical Qualification
PCNs	Primary Care Networks
IMG	International Medical Graduate
NWRS	National Workforce Reporting Service
DPC	other Direct Patient Care roles (i.e., non-GP and non-nurse)

Supplementary Figures 1a & 1b - Relationship between data sources

Relationship between data sources for (a) General Practitioners and (b) Specialist Doctors. Circles are not to proportional size. Note, a small number of doctors are both on the GMC GP and Specialist Register (1,045 on 27 March 2025) and therefore may be working both as a GP and Consultant.

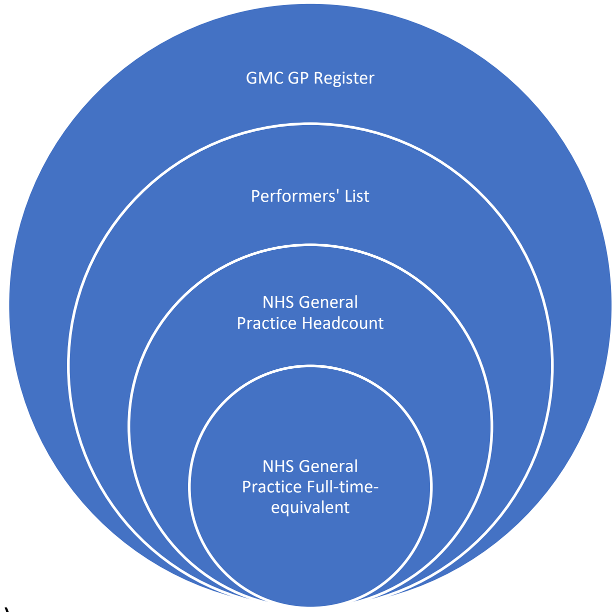

1a)

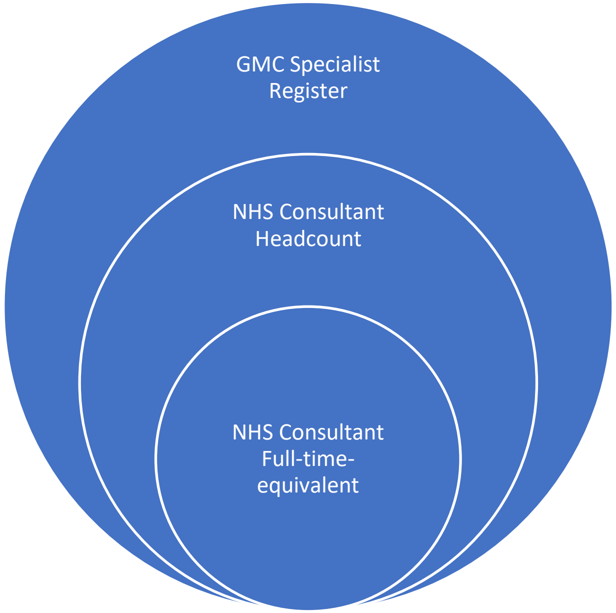

1b)

Supplementary Table 1 - Data on general practitioners available

Data on general practitioners in England available in data sources

**Name (source)**	**GP Register (General Medical Council)**	**England's GP Performers' List (Primary Care Support England)**	**General Practice Workforce dataset (NHS England)**
Included population	• All doctors eligible for appointment as a GP in the UK, registered:<br>o with a licence to practise<br>o without a licence to practise	• All GPs approved to provide care in an NHS or Armed Services setting in England by the following roles:<br>o GP performer<br>o GP contractor (i.e., GP partner- this category is underutilised, and most GPs are listed are performers)<br>o GP registrar (apply to join the Performers' List three to six months before completing GP training)<br>o Armed Services Type 1 & 2 GPs<br>• Can filter by trainees, those included with conditions, suspended or on probation	• GPs working in NHS general practice in England by the following roles:<br>o GP partner<br>o Salaried GP<br>o Long-term GP locum (e.g., covering job vacancy)<br>o GP retainer (GPs supported to return to work after a career break)<br>o GP in training grade (include Foundation Years doctors F1 and F2, GP Specialist Trainees ST1-4, other training grades)
Excluded population	• GP trainees (data is reported by the GMC elsewhere, see below).<br>• GPs no longer on the GP register (e.g., relinquished registration, deceased, removed for administrative reasons, erased after fitness to practise hearing).<br>• < 1% of all doctors are excluded from the GMC's 'Data Explorer' for data protection purposes.	• GPs who have not applied to provide care in an NHS or Armed Services setting in England.<br>• GPs who have been removed from the Performer's List.	• Ad hoc GP locums, e.g., short-term holiday cover (reported by NHSE in supplementary 'General Practice Workforce Bulletin' annex tables).<br>• GPs working in Prisons, Army Bases, Educational Establishments, Specialist Care Centres including Drug Rehabilitation Centres, Walk-In Centres and other alternative settings.<br>• GPs employed via Primary Care Network funding
Data source(s)	• Individual GPs who must pay an annual retention fee to remain on the register.<br>• Every five years to re-licence a 'Responsible Officer' from NHS England or the 'Independent Doctors Federation', if only working privately,	• Individual GPs when they first register to join the list three to six months before completing GP training.<br>• GPs can update their personal and employment details via the PCSE website. The employing practice can also log in to amend these details	• NHS general practices, via a nominated person, submit details about the practice's workforce through the National Workforce Reporting Service (NWRS) online portal. This is a contractual and legal requirement as part of the Workforce Minimum Dataset (wMDS) collection. However, on average 1.1% of GPs by HC and 2.2% of FTEs

Name (source)	GP Register (General Medical Council)	England's GP Performers' List (Primary Care Support England)	General Practice Workforce dataset (NHS England)
	recommends a GP for revalidation by the GMC if they have met the necessary annual appraisal requirements.	• Employment details include the name of the GP practice and whether a GP is salaried or principal (i.e., partner).	(including trainees) were estimated due to missing data between September 2015 and August 2024. Estimates were higher when the time series began (e.g., 4.9% of GP HC and 7.8% of FTEs were estimated in September 2015).<br>• From June 2018, GP trainee data have been taken from the Health Education England (HEE) Trainee Information System (TIS) and the previous time series was revised to capture this.
Public availability of statistics	Via the GMC's 'The Medical Register' (online search tool)<br>• GPs' name, gender, provisional and full registration date, date added to the GP register, place and year of primary medical qualification, NHSE regional team (designated body) and named Responsible Officer +/- details of conditions, warnings or suspensions to practise.<br><br>Via the GMC's 'Data Explorer' (online dashboard) of 'The Medical Register'<br>• Total registered GP HC: with or without license to practise; if also on the specialist register; place and year of primary medical qualification.<br><br>Via the GMC's 'State of Medical Education and Practice (SoMEP) in the UK: Workforce Report 2024' excel reference tables based on 'The Medical Register'<br>• Total registered licensed GP HC: by UK country, England region, age,	Via the full 'Performers' List' (online search or excel download)<br>• GPs' name, GMC number, date of registration as a doctor, date first on performers' list, date on GP register, NHSE regional team, performer role (contractor, performer, registrar) +/- details of conditions, suspensions, if on probation or if on 24-hour retirement (to draw down pension benefits before returning to work).	Via NHS England's General Practice Workforce series of Official Statistics (CSV download or General Practice Workforce Interactive Dashboard, and Bulletin Tables publish national data as excel file)<br>Individual GP level data (GPs are anonymised)<br>• FTE, role, gender, age, age band, country area and group of primary medical qualification, Integrated Care Board, NHS England region<br>Practice level data<br>• Practice, number of registered patients at the practice (by age bands and gender), Primary Care Network, Integrated Care Board, NHS England region<br>• Total GP HC by role, gender, age bands, country area and group of primary medical qualification<br>• Total FTE GP by role, gender<br>National Level data (Bulletin Tables)<br>• Gender and role (FTE and HC), age band (FTE and HC), work commitment (HC), country of qualification an role (HC), NHS England region count by role (FTE and HC), NHS England region count by ethnicity (HC) (Annex A: percentages of general practices providing data; Annex B-C: ad-hoc locums at national and regional (FTE and HC))

Name (source)	GP Register (General Medical Council)	England's GP Performers' List (Primary Care Support England)	General Practice Workforce dataset (NHS England)
	gender, PMQ, ethnicity, religion, sexual orientation, disability		
Frequency of updates	• 'Medical Register': Daily<br>• 'Data Explorer' time series: Annual<br>• 'SoMEP' workforce report: Annual	• Daily	• Monthly since June 2021.
Time series data trends publicly available	• 'Medical Register': time series data not publicly available<br>• 'Data Explorer': annual from 31st December 2006<br>• 'SoMEP' workforce report reference tables: annual 31 December 2012 – 31 December 2023 for 2024 report	• Time series data not publicly available	• Quarterly from 30th September 2015, monthly from 30th June 2021 (no data in December 2015 and June 2016).

Key: GP=General Practitioner, GMC=General Medical Council, PCSE=Primary Care Support England, NHSE=NHS England, HC=Headcount, FTE=full-time-equivalent (37.5 hours per week), PMQ= place of Primary Medical Qualification, SoMEP: State of Medical Education and Practice

Supplementary Table 2 - Total number of general practitioners and specialist doctors

Total number of GMC-licensed GPs; GPs on the Performers' List; fully qualified GPs in NHS general practice (HC and FTE); GMC-licensed specialists; NHS Consultants reported in NHS Trust and other core NHS organisations (excluding primary care) (HC and FTE) – number at start and end of time series, percentage change, change per year linear regression coefficient, 95% confidence intervals, and number (%) not in data source in 2015 and 2024.

Data Source	Time-series	Total at start of time series	Total at end of time series	% change between start and end of time series	Change per year linear regression coefficient	95% confidence interval	Number (%) of GMC licensed doctors not in data source 2015	Number (%) of GMC licensed doctors not in data source 2024	Number (%) of GPs on Performers' List not in data source 2024
GMC Licensed GPs	2012-2024	48,586	58,548	21%	825/year	712 to 939			
GMC Licensed GPs	2015-2024	49,574	58,548	18%	985/year	884 to 1086			
Performer's List GPs	2024		55,958					2,590 (4%)	
NHS General Practice GPs	2015-2024	36,082	38,626	7%	207/year	82 to 332	13,492 (27%)	19,922 (34%)	17,332 (31%)
NHS General Practice GPs FTE	2015-2024	29,364	28,197	-4%	-199/year	-295 to -102	20,210 (41%)	30,351 (52%)	27,761 (50%)
GMC Licensed Specialists	2012-2024	54,340	77,299	42%	1,846/year	1,757 to 1,936			
GMC Licensed Specialists	2015-2024	59,172	77,299	31%	1,948/year	1,832 to 2,063			
NHS Consultants	2015-2024	45,655	63,244	39%	1,929/year	1,821 to 2,037	13,517 (23%)	14,055 (18%)	
NHS Consultants FTE	2015-2024	43,176	58,382	35%	1,674/year	1,602 to 1,745	15,996 (27%)	18,917 (24%)	

Supplementary Table 3 - Full-time-equivalent to headcount proportions

Full-time-equivalent to headcount proportion; NHS consultants, fully qualified GPs in NHS General Practice, fully qualified GPs in NHS General Practice by gender & age.

Year	NHS Consultant FTE/HC	NHS Total GP FTE/HC	NHS Female GP FTE/HC	NHS Male GP FTE/HC	NHS unknown / other gender GP FTE/HC	NHS <30 GP FTE/HC	NHS 30-39 GP FTE/HC	NHS 40-49 GP FTE/HC	NHS 50-59 GP FTE/HC	NHS 60+ GP FTE/HC	NHS unknown age GP FTE/HC
2015	0.95	0.81	0.72	0.91	0.88	0.81	0.78	0.81	0.85	0.77	0.86
2016	0.94	0.82	0.72	0.91	1.07	0.77	0.77	0.81	0.85	0.79	0.99
2017	0.94	0.79	0.70	0.89	0.73	0.71	0.75	0.79	0.84	0.79	0.69
2018	0.94	0.78	0.70	0.88	0.73	0.72	0.73	0.78	0.83	0.78	0.67
2019	0.94	0.76	0.68	0.87	0.73	0.71	0.71	0.76	0.82	0.77	0.70
2020	0.94	0.75	0.67	0.85	0.72	0.69	0.70	0.75	0.80	0.76	0.70
2021	0.94	0.75	0.67	0.85	0.71	0.74	0.70	0.75	0.80	0.76	0.67
2022	0.93	0.75	0.68	0.84	0.71	0.76	0.70	0.74	0.79	0.76	0.69
2023	0.93	0.74	0.67	0.84	0.70	0.73	0.69	0.73	0.79	0.76	0.68
2024	0.92	0.73	0.66	0.82	0.70	N/A	N/A	N/A	N/A	N/A	N/A
Absolute FTE change between first & final year	-0.02	-0.08	-0.06	-0.08	-0.18	-0.09	-0.08	-0.08	-0.06	-0.01	-0.18
% Change between first & final year	-2%	-10%	-8%	-9%	-20%	-11%	-11%	-9%	-7%	-1%	-21%

Supplementary Figure 2 - NHS registered patients-per-doctor

NHS registered patients per general practitioner and per specialist doctor in England

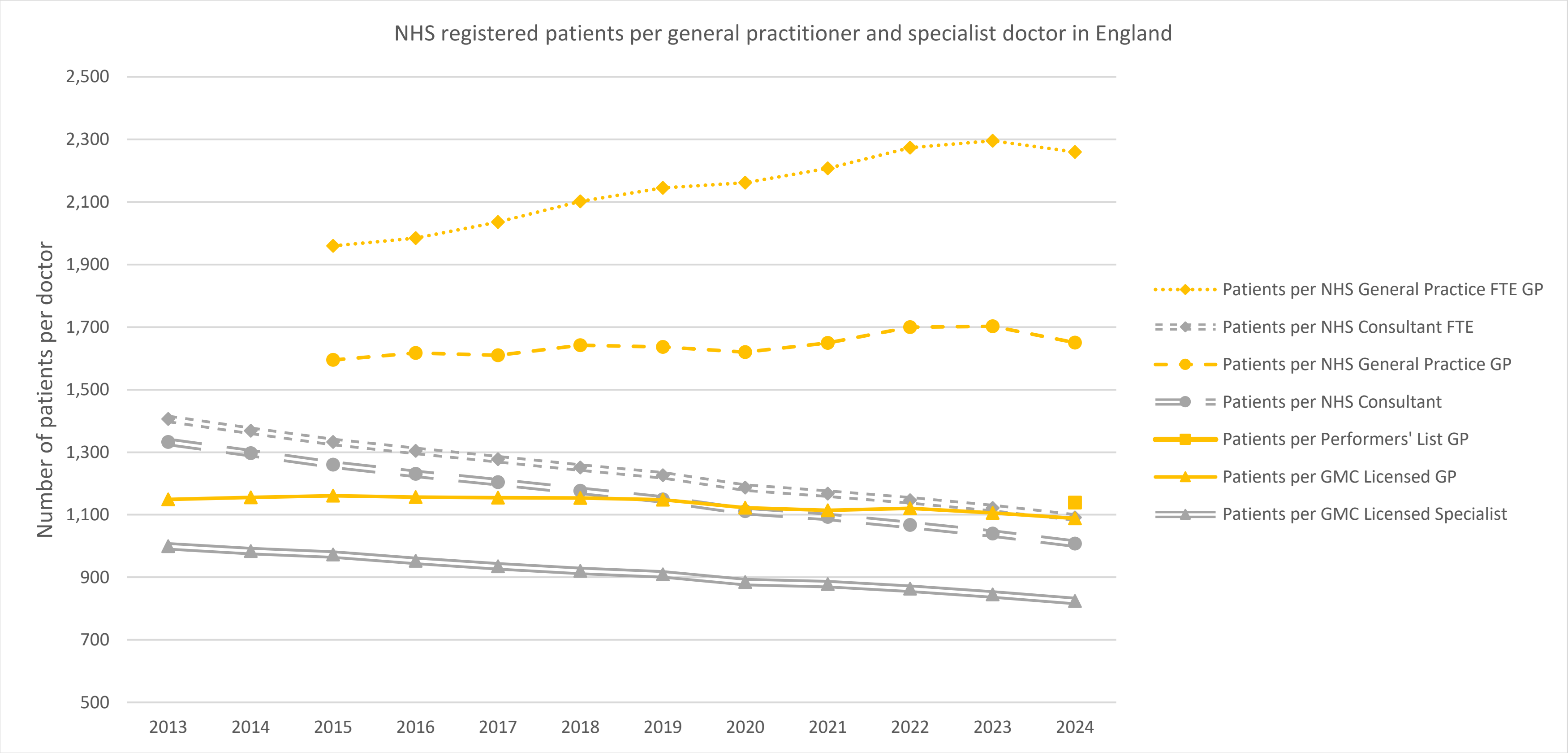

Supplementary Table 4 - NHS registered patients-per-doctor

NHS registered patients-per-doctor; number at the start and end of time series, percentage change, change per year linear regression coefficient, 95% confidence intervals.

Data Source	Time-series	Patients-per-doctor at start of time series	Patients-per-doctor at end of time series	% change between start and end of time series*	Change per year linear regression coefficient	95% confidence interval
GMC Licensed GPs	2015-2024	1161	1088	-6%	-7.97	-10.02 to -5.92
GMC Licensed Specialists	2015-2024	972	824	-15%	-15.71	-16.68 to -52.93
NHS General Practice GPs	2015-2024	1595	1650	3%	9.37	3.53 to 15.21
NHS Consultants	2015-2024	1260	1008	-20%	-27.81	-28.73 to -26.90
NHS General Practice GPs FTE	2015-2024	1960	2260	15%	38.84	31.76 to 45.93
NHS Consultants FTE	2015-2024	1333	1092	-18%	-26.62	-27.75 to -25.48
Performer's List GPs	2024		1139			

Supplementary Table 5 - General Practitioners by gender

GMC-licensed GPs and fully qualified GPs reported in NHS general practice (HC and FTE) in England by gender – total number at start and end of time series, percentage change, change per year linear regression coefficient, 95% confidence intervals; and number (%) of GMC-licensed GPs not in NHS general practice in 2015 and 2024

Data source	Gender	Time-series	Total at start of time series	Total at end of time series	% change	Change per year linear regression coefficient	95% confidence interval	Number (%) of GMC licensed GPs not in NHS general practice 2015	Number (%) of GMC licensed GPs not in NHS general practice 2024
GMC Licensed	Female	2012-2024	23,211	33,555	45%	870	844 to 896		
GMC Licensed	Female	2015-2024	25,462	33,555	32%	892	854 to 931		
NHS General Practice	Female	2015-2024	17,844	22,340	25%	438	348 to 528	7,618 (30%)	11,215 (33%)
NHS General Practice FTE	Female	2015-2024	12,831	14,803	15%	178	138 to 218	12,631 (50%)	18,752 (56%)
GMC Licensed	Male	2012-2024	25,375	24,993	-2%	-45	-144 to 54		
GMC Licensed	Male	2015-2024	24,112	24,993	4%	92	0 to 185		
NHS General Practice	Male	2015-2024	16,604	15,994	-4%	-135	-203 to -67	7,508 (31%)	8,999 (36%)
NHS General Practice FTE	Male	2015-2024	15,096	13,183	-13%	-282	-350 to -215	9,016 (37%)	11,810 (47%)
NHS General Practice	Other / unknown	2015-2024	1,640	302	-82%	-97	-172 to -21		
NHS General Practice FTE	Other / unknown	2015-2024	1,437	211	-85%	-95	-162 to -27		

Supplementary Table 6a - General Practitioners by age-band and gender

GMC-licensed GPs and fully qualified GPs reported in NHS general practice in England by age-band and gender – percentage change, change per year linear regression coefficient, 95% confidence intervals; and number (%) of GMC-licensed GPs not in NHS general practice in 2015 and 2023

Data source	Age-band	Gender	Time-series	Start of time series	End of time series	% change	Change per year linear regression coefficient	95% confidence interval (lower)	95% confidence interval (higher)	Number and % of GMC licensed GPs not in NHS general practice 2015	Number and % of GMC licensed GPs not in NHS general practice 2023
GMC Licensed	<30	F	2012-2023	476	175	-63%	-33.24	-41.62	-24.86		
GMC Licensed	<30	F	2015-2023	522	175	-66%	-43.60	-49.61	-37.59		
NHS General Practice	<30	F	2015-2023	363	128	-65%	-22.65	-33.85	-11.45	159 (30%)	47 (27%)
NHS General Practice FTE	<30	F	2015-2023	295	103	-65%	-16.78	-27.26	-6.30	227 (43%)	72 (41%)
GMC Licensed	30-39	F	2012-2023	8265	10604	28%	230.99	176.02	285.95		
GMC Licensed	30-39	F	2015-2023	9192	10604	15%	166.08	90.11	242.06		
NHS General Practice	30-39	F	2015-2023	6268	6640	6%	58.90	-22.42	140.22	2924 (32%)	3964 (37%)
NHS General Practice FTE	30-39	F	2015-2023	4434	4405	-1%	-0.34	-39.00	38.32	4758 (52%)	6199 (58%)
GMC Licensed	40-49	F	2012-2023	7654	12018	57%	406.01	355.17	456.86		
GMC Licensed	40-49	F	2015-2023	8286	12018	45%	480.68	440.50	520.86		
NHS General Practice	40-49	F	2015-2023	6076	7955	31%	239.55	208.85	270.25	2210 (27%)	4063 (34%)
NHS General Practice FTE	40-49	F	2015-2023	4312	5251	22%	113.92	103.07	124.77	3974 (48%)	6767 (56%)
GMC Licensed	50-59	F	2012-2023	5241	7354	40%	180.13	166.85	193.42		
GMC Licensed	50-59	F	2015-2023	5947	7354	24%	166.63	147.92	185.35		
NHS General Practice	50-59	F	2015-2023	4264	5124	20%	86.75	58.77	114.73	1683 (28%)	2230 (30%)
NHS General Practice FTE	50-59	F	2015-2023	3163	3605	14%	36.36	15.63	57.08	2784 (47%)	3749 (51%)
GMC Licensed	60+	F	2012-2023	1575	2368	50%	81.49	55.41	107.56		
GMC Licensed	60+	F	2015-2023	1515	2368	56%	119.73	100.76	138.71		

NHS General Practice	60+	F	2015-2023	749	1289	72%	62.77	56.49	69.04	766 (51%)	1079 (46%)
NHS General Practice FTE	60+	F	2015-2023	540	889	65%	38.93	32.80	45.06	975 (64%)	1479 (62%)
NHS General Practice	Unknown	F	2015-2023	123	92	-25%	-6.45	-16.43	3.53		
NHS General Practice FTE	Unknown	F	2015-2023	87	55	-37%	-5.48	-11.84	0.87		
GMC Licensed	<30	M	2012-2023	253	121	-52%					
GMC Licensed	<30	M	2015-2023	236	121	-49%	-16.32	-19.76	-12.87		
NHS General Practice	<30	M	2015-2023	169	76	-55%	-11.53	-15.45	-7.62	67 (28%)	45 (37%)
NHS General Practice FTE	<30	M	2015-2023	144	70	-52%	-8.21	-12.67	-14.59	92 (39%)	51 (43%)
GMC Licensed	30-39	M	2012-2023	5350	6256	17%					
GMC Licensed	30-39	M	2015-2023	5506	6256	14%	81.35	19.12	143.58		
NHS General Practice	30-39	M	2015-2023	3729	3541	-5%	-19.55	-42.20	3.10	1777 (32%)	2715 (43%)
NHS General Practice FTE	30-39	M	2015-2023	3369	2734	-19%	-82.00	-108.76	-55.24	2137 (39%)	3522 (56%)
GMC Licensed	40-49	M	2012-2023	6667	7413	11%					
GMC Licensed	40-49	M	2015-2023	6448	7413	15%	121.93	99.84	144.02		
NHS General Practice	40-49	M	2015-2023	4688	4911	5%	27.55	-6.13	61.23	1760 (27%)	2502 (34%)
NHS General Practice FTE	40-49	M	2015-2023	4407	4211	-4%	-31.24	-57.91	-4.58	2041 (32%)	3202 (43%)
GMC Licensed	50-59	M	2012-2023	7953	6151	-23%					
GMC Licensed	50-59	M	2015-2023	7599	6151	-19%	-195.88	-235.11	-156.66		
NHS General Practice	50-59	M	2015-2023	5658	4345	-23%	-189.00	-216.72	-161.28	1941 (26%)	1806 (29%)
NHS General Practice FTE	50-59	M	2015-2023	5312	3886	-27%	-209.11	-242.32	-175.89	2287 (30%)	2265 (37%)
GMC Licensed	60+	M	2012-2023	5152	4604	-11%					
GMC Licensed	60+	M	2015-2023	4323	4604	7%	56.45	26.69	86.21		
NHS General Practice	60+	M	2015-2023	2219	2499	13%	24.90	4.25	45.55	2104 (49%)	2105 (46%)
NHS General Practice FTE	60+	M	2015-2023	1755	1999	14%	17.61	-2.55	37.78	2568 (59%)	2605 (57%)
NHS General Practice	Unknown	M	2015-2023	137	68	-50%	-10.83	-17.75	-3.92		
NHS General Practice FTE	Unknown	M	2015-2023	108	45	-58%	-8.94	-13.72	-4.15		

Supplementary Table 6b - General Practitioners by age-band only

GMC-licensed GPs and fully qualified GPs reported in NHS general practice in England by age-band – total number at start end of time series, percentage change, change per year linear regression coefficient, 95% confidence intervals; number (%) of GMC-licensed GPs not in NHS general practice in 2015 and 2023

Data source	Age band	Time-series	Total at start of time series	Total at end of time series	% change	Change per year linear regression coefficient	95% confidence interval	Number(%) of GMC licensed GPs not in NHS general practice 2015	Number (%) of GMC licensed GPs not in NHS general practice 2023
GMC Licensed	<30	2012-2023	729	296	-59%	-47	-57 to -37		
GMC Licensed	<30	2015-2023	758	296	-61%	-60	-68 to - 52		
NHS General Practice	<30	2015-2023	542	238	-56%	-30	-45 to -15	216 (28%)	58 (20%)
NHS General Practice FTE	<30	2015-2023	441	173	-61%	-25	-40 to -11	317 (42%)	123 (42%)
GMC Licensed	30-39	2012-2023	13,615	16,860	24%	284	257 to 311		
GMC Licensed	30-39	2015-2023	14,698	16,860	15%	247	217 to 278		
NHS General Practice	30-39	2015-2023	10,016	10,284	3%	48	-32 to 129	4,682 (32%)	6,576 (39%)
NHS General Practice FTE	30-39	2015-2023	7,806	7,141	-9%	-83	-111 to -55	6,892 (47%)	9,719 (58%)
GMC Licensed	40-49	2012-2023	14,321	19,431	36%	494	422 to 566		
GMC Licensed	40-49	2015-2023	14,734	19,431	32%	603	5684to 641		
NHS General Practice	40-49	2015-2023	10,772	12,893	20%	269	207 to 331	3,962 (27%)	6,538 (34%)
NHS General Practice FTE	40-49	2015-2023	8,722	9,468	9%	84	49 to 119	6,012 (41%)	9,963 (51%)
GMC Licensed	50-59	2012-2023	13,194	13,505	2%	-11	-424to 22		
GMC Licensed	50-59	2015-2023	13,546	13,505	0%	-29	-85 to 25		
NHS General Practice	50-59	2015-2023	9,924	9,473	-5%	-102	-154 to - 51	3,622 (27%)	4,032 (30%)
NHS General Practice FTE	50-59	2015-2023	8,477	7,492	-12%	-173	-226 to -121	5,069 (37%)	6,013 (45%)
GMC Licensed	60+	2012-2023	6,727	6,972	4%	55	-25 to 135		
GMC Licensed	60+	2015-2023	5,838	6,972	19%	176	128 to 224		
NHS General Practice	60+	2015-2023	2,970	3,790	28%	88	66 to 110	2,868 (49%)	3.182 (46%)
NHS General Practice FTE	60+	2015-2023	2,297	2,889	26%	57	34 to 79	3,541 (61%)	4,083 (59%)
NHS General Practice	Unknown	2015-2023	1,881	475	-75%	-128	-221 to -35		
NHS General Practice FTE	Unknown	2015-2023	1,620	324	-80%	-124	-210 to -37		

Supplementary Figure 3 - General Practitioners by age-band

GMC-licensed GPs and fully qualified GPs reported in NHS general practice by headcount and FTE in England by age-band

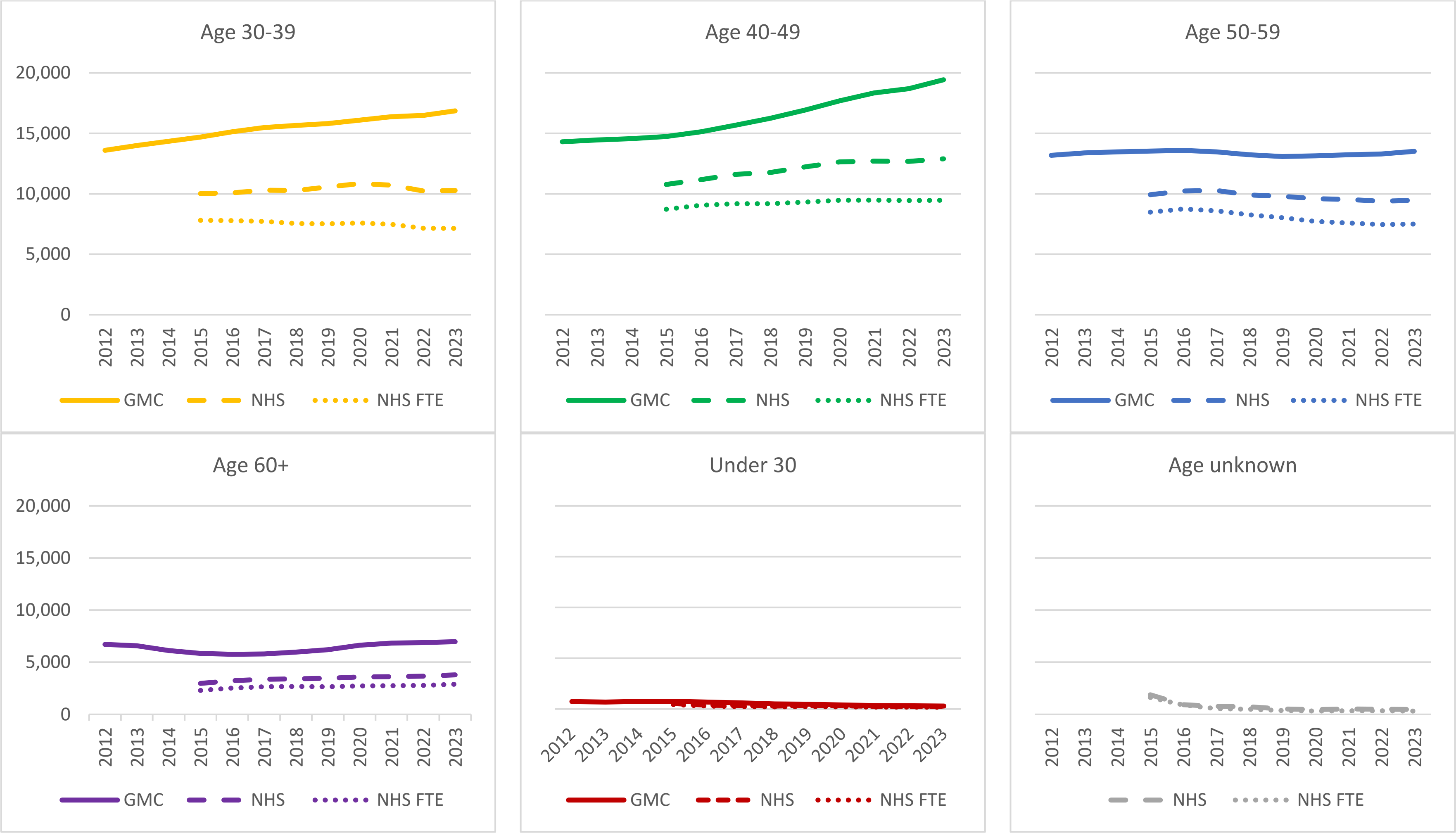

GMC=General Medical Council Licensed GPs, NHS=GPs working in NHS General Practice, FTE= full-time-equivalent

Supplementary Table 7a - General Practitioner proportion by place of primary medical qualification

Proportion of GPs by place of primary medical qualification (PMQ) out of all GPs in GMC and NHS general practice data sources (FTE data not available)

Year	GMC - Proportion of place of PMQ from UK	GMC - Proportion of place of PMQ from non-UK country	NHS - Proportion of place of PMQ from UK	NHS - Proportion of place of PMQ from non-UK country	NHS - Proportion of place of PMQ unknown
2012	0.76	0.24			
2013	0.76	0.24			
2014	0.76	0.24			
2015	0.76	0.24	0.68	0.19	0.13
2016	0.77	0.23	0.74	0.21	0.06
2017	0.77	0.23	0.73	0.20	0.06
2018	0.77	0.23	0.73	0.20	0.07
2019	0.77	0.23	0.71	0.19	0.10
2020	0.77	0.23	0.71	0.19	0.10
2021	0.77	0.23	0.73	0.20	0.07
2022	0.76	0.24	0.74	0.21	0.05
2023	0.75	0.25	0.74	0.22	0.04
2024	0.74	0.26	0.73	0.23	0.03
Average	**0.76**	**0.24**	**0.73**	**0.20**	**0.07**

Supplementary Table 7b - General Practitioners by place of primary medical qualification

GMC-licensed GPs and fully qualified GPs reported in NHS general practice in England by place of primary medical qualification – number, percentage change, change per year linear regression coefficient, 95% confidence intervals; number (%) of GMC-licensed GPs not in NHS general practice in 2015 and 2024

Data source	PMQ	Time-series	Total at start of time series	Total at nd of time series	% change between start and end of time series	Change per year linear regression coefficient	95% confidence interval	Number (%) of GMC licensed GPs not in NHS general practice 2015	Number (%) of GMC licensed GPs not in NHS general practice 2024
GMC Licensed	UK	2012-2024	36,960	43,308	17%	585	530 to 640		
GMC Licensed	UK	2015-2024	37,872	43,308	14%	613	528 to 698		
NHS General Practice	UK	2015-2024	24,717	28,481	15%	258	104 to 412	13,155 (35%)	14,827 (34%)
GMC Licensed	non-UK	2012-2024	11,626	15,240	31%	241	128 to 354		
GMC Licensed	non-UK	2015-2024	11,702	15,240	30%	372	223 to 520		
NHS General Practice	non-UK	2015-2024	6,973	9,072	30%	155	35 to 274	4,729 (40%)	6,168 (40%)
NHS General Practice	Unknown	2015-2024	4,595	1,271	-72%	-219	-443 to 6		

Supplementary Table 8 - Patients-per-FTE general practitioner by region

NHS registered patients per GMC-licensed GP and fully qualified GP reported in NHS general practice in England by region – number and percentage of patients per GMC-licensed GPs not in NHS general practice on 31 December 2024

Source	Region	Patients per GP	Difference between number of patients per GP between GMC and NHS in 2024	% difference between number of patients per GP between GMC and NHS in 2024
GMC	North West	1,049		
NHS General Practice	North West	1,622	572	55%
NHS General Practice FTE	North West	2,181	1,131	108%
GMC	North East and Yorkshire	1,119		
NHS General Practice	North East and Yorkshire	1,585	466	42%
NHS General Practice FTE	North East and Yorkshire	2,150	1,031	92%
GMC	Midlands	1,158		
NHS General Practice	Midlands	1,694	536	46%
NHS General Practice FTE	Midlands	2,237	1,079	93%
GMC	East of England	1,234		
NHS General Practice	East of England	1,803	570	46%
NHS General Practice FTE	East of England	2,400	1,166	95%
GMC	London	1,074		
NHS General Practice	London	1,787	713	66%
NHS General Practice FTE	London	2,496	1,422	132%
GMC	South West	923		
NHS General Practice	South West	1,381	458	50%
NHS General Practice FTE	South West	1,984	1,061	115%
GMC	South East	1,057		
NHS General Practice	South East	1,646	589	56%
NHS General Practice FTE	South East	2,321	1,264	120%

Supplementary Table 9 - Full-time-equivalent to headcount proportion by region

Full-time-equivalent to headcount proportion in England by region on 31 December 2024

	NHS East of England	NHS London	NHS Midlands	NHS North East and Yorkshire	NHS North West	NHS South East	NHS South West
FTE/HC GP proportion	0.75	0.72	0.76	0.74	0.74	0.71	0.70
